# The prognostic performance of qSOFA for community-acquired pneumonia

**DOI:** 10.1186/s40560-018-0307-7

**Published:** 2018-08-08

**Authors:** Fumiaki Tokioka, Hiroshi Okamoto, Akio Yamazaki, Akihiro Itou, Tadashi Ishida

**Affiliations:** 1 Department of Respiratory Medicine, Kurashiki Central Hospital, 1-1-1 Miwa, Kurashiki, Okayama, 710-8602 Japan; 2 Center for Clinical Epidemiology, St. Luke’s International University, Tokyo, Japan

**Keywords:** qSOFA, CURB-65, PSI, Pneumonia, Sepsis

## Abstract

**Background:**

Quick Sepsis-related Organ Failure Assessment (qSOFA) is a new screening system for sepsis. The prognostic performance of qSOFA for patients with suspected infections outside the intensive care unit (ICU) is similar to that of full SOFA; however, its performance for community-acquired pneumonia (CAP) has not yet been evaluated in detail.

The objectives of the present study were to compare the prognostic performance of qSOFA with existing pneumonia severity scores, such as CURB-65 (confusion, blood urea nitrogen > 19 mg/dL, respiratory rate ≥ 30/min, systolic blood pressure < 90 mmHg, or diastolic blood pressure ≤ 60 mmHg, age ≥ 65 years) and the pneumonia severity index (PSI), and examine its usefulness for predicting mortality and ICU admission in patients with CAP of high severity and mortality that requires hospitalization.

**Methods:**

We performed a secondary analysis of data from a prospective observational study of adult patients who were admitted to our hospital between October 2010 and June 2016. We compared the prognostic performance of qSOFA, CURB-65, and PSI for predicting in-hospital mortality and ICU admission using the *C* statistics.

**Results:**

The median age of the 1045 enrolled patients was 77 (68–83) years, and 71.4% were males. The in-hospital mortality and ICU admission rates of the entire cohort were 6.1 and 7.9%, respectively. All scores were significantly higher in non-survivors and ICU admission patients than in survivors and non-ICU admission patients (*p* < 0.001). The *C* statistics of qSOFA for predicting in-hospital mortality was 0.69 (95% CI 0.63–0.75), and no significant differences were observed between CURB-65 (*C* statistics, 0.75; 95% CI 0.69–0.81) and PSI (*C* statistics, 0.74; 95% CI 0.69–0.80). The *C* statistics of qSOFA for predicting ICU admission was 0.76 (95% CI 0.71–0.80), and no significant differences were noted between CURB-65 (*C* statistics, 0.73; 95% CI 0.67–0.79) and PSI (*C* statistics, 0.72; 95% CI 0.66–0.78).

**Conclusions:**

Regarding hospitalized CAP, the prognostic performance of qSOFA for in-hospital mortality and ICU admission was not significantly different from those of CURB-65 and PSI. qSOFA only requires a few items and vital signs, and, thus, may be particularly useful for emergency department or non-respiratory specialists.

**Electronic supplementary material:**

The online version of this article (10.1186/s40560-018-0307-7) contains supplementary material, which is available to authorized users.

## Background

Sepsis-3, a new definition for sepsis, and its diagnostic criteria were published in 2016 [1]. Quick Sepsis-related Organ Failure Assessment (qSOFA) has been used to screen sepsis outside of the intensive care unit (ICU) [2]. Sepsis is diagnosed if at least two out of the three criteria are positive in patients with suspected infections. qSOFA is a very simple screening tool because it only has three evaluation items, does not require laboratory examinations, and may be conducted at the bedside. Moreover, in the original study, the *C* statistics for the in-hospital mortality of qSOFA was 0.81 as a good indicator of prognosis, and its usefulness as a prognostic tool has been evaluated [3–5]; however, some studies have questioned its usefulness [6–8].

Community-acquired pneumonia (CAP) is a common infection, is frequently a causative disease for sepsis, and its mortality rate is between approximately 3 and 9% [1, 2, 9–13]. Therefore, the prognosis of CAP needs to be accurately assessed in order to select an appropriate treatment strategy.

Few studies have examined the use of qSOFA for CAP [3, 10, 12]; however, one of these studies reported a high mortality rate [3], while another was a short report in a letter format [10]. Thus, there is currently no consensus as to whether qSOFA is an effective strategy.

A previous study comprehensively examined existing pneumonia severity scores for CAP and qSOFA [12]; however, relatively mild pneumonia that may be treated internally was incorporated and the patient background used was relatively young. The original clinical significance of severity scores was for pneumonia of high severity and mortality that requires hospitalization; however, it currently remains unclear whether qSOFA is useful as a prognostic tool for CAP that requires hospitalization.

The aims of the present study were to compare the prognostic accuracy of qSOFA with existing pneumonia severity scores (CURB-65 [Confusion, Urea, Respiratory Rate, Blood Pressure, and Age] [11] and the pneumonia severity index [PSI] [14]) and to examine the usefulness of qSOFA for CAP of high severity and mortality that requires hospitalization.

## Methods

### Study design and settings

We performed a secondary analysis of data from a prospective observational study of patients admitted to Kurashiki Central Hospital (1166-bed community hospital, Okayama, Japan) between October 2010 and June 2016.

Adult patients (age, ≥ 18 years) with pneumonia diagnosed by new infiltrates on chest imaging studies (radiography or computed tomography) and two or more symptoms consistent with pneumonia (including cough, dyspnea, fever, sputum production, breathlessness, and/or pleuritic chest pain) were enrolled at an emergency department or outpatient visit.

Exclusion criteria were as follows: age younger than 18 years, a resident of an extended care facility or nursing home, recently discharged from hospital within 90 days, an elderly or disabled individual receiving nursing care, and those receiving regular endovascular treatments as an outpatient (dialysis, antibiotic therapy, chemotherapy, and immunosuppressant therapy).

The present study was approved by the Kurashiki Central Hospital Ethics Committee. Informed consent was obtained from all patients at the time of admission.

### Data collection

Data on demographic characteristics, vital signs, imaging, and laboratory test results of the enrolled patients were recorded upon hospital arrival at an emergency department or outpatient visit. All patients who were intubated immediately after hospital arrival also recorded vital signs before intubation. qSOFA, CURB-65, and PSI were calculated using data obtained at enrollment. All data were collected by a study team consisting of a four board-certificated pulmonologist.

### Outcome variables

The primary outcome was in-hospital mortality and the secondary outcome was ICU admission.

### Statistical analysis

We assessed the predictive performance of qSOFA, CURB-65, and PSI for the primary and secondary outcomes.

Data were presented as medians with interquartile ranges for continuous variables and as numbers and percentages for categorical variables. Variables with significant differences were tested by a binary logistic analysis together with the qSOFA score and presented with an odds ratio (OR) with a 95% confidence interval (CI). Categorical variables were compared using χ2 statistics. In order to assess the discriminatory power of the qSOFA score for predicting outcomes, we compared the *C* statistics of the qSOFA score with those of the CURB-65 and PSI scores. The *C* statistic is a summary measure of discrimination which quantifies the ability of the model to assign a high probability. *C* statistics are equivalent to the area under the receiver operating characteristic curve. *C* statistics range from 0.5 to 1.0; a measure of 0.5 indicates that the discrimination is caused by chance alone, and 1.0 indicates perfect discrimination. All *p* value analyses were two-sided and a *p* value of less than 0.05 was considered to be significant. All statistical analyses were performed with EZR (Saitama Medical Center, Jichii Medical University, Saitama, Japan), which is a graphical user interface for R (The R Foundation for Statistical Computing, Vienna, Austria). More precisely, it is a modified version of R commander designed to add statistical functions frequently used in biostatistics [15].

## Results

### Characteristics of the study cohort

A total of 1954 patients were evaluated in the enrollment period, and 909 were excluded, among which three were < 18 years, 133 were residents of an extended care facility or nursing home, 361 were recently discharged from hospital within 90 days, 301 were elderly or disabled and receiving nursing care, and 111 were receiving regular endovascular treatment as outpatients. Some patients fulfilled multiple items.

We ultimately enrolled 1045 patients with pneumonia, and their baseline characteristics are listed in Table 1. The median age of these patients was 77 (68–83) years, and 71.4% were males. The main comorbidities were respiratory diseases, such as chronic obstructive pulmonary disease (COPD) (25.5%) and bronchial asthma (13.8%), diabetes (19.5%), chronic heart failure (30.9%), and cerebrovascular diseases (14.3%). The median of qSOFA was 1 point (0–1), the median of CURB-65 was 2 points (1–2), and the median score of PSI was 97 (81–120) and its median class was class IV (III-IV).

**Table 1:** Baseline characteristics of the study cohort

		In-hospital mortality			ICU admission		
Variables	All encounters	Non-survivors	Survivors	*P* value	ICU admission	Non-ICU	*P* value
Number of patients	1045	64 (6.1)	981 (93.9)		83 (7.9)	962 (92.1)	
Age, years	77 (68–83)	80 (74–85)	76 (68–83)	0.004	74 (66–82)	77 (68–84)	0.046
Male sex, %	746 (71.4)	51 (79.7)	697 (70.8)	0.15	64 (77.1)	682 (70.9)	0.26
Comorbidities, %							
COPD	266 (25.5)	26 (40.6)	240 (24.5)	0.007	24 (28.9)	242 (25.2)	0.51
Interstitial pneumonia	73 (7.0)	6 (9.4)	67 (6.8)	0.44	3 (3.6)	70 (7.3)	0.27
Old pulmonary tuberculosis	37 (3.5)	6 (9.4)	31 (3.2)	0.022	4 (4.8)	33 (3.4)	0.53
Asthma	144 (13.8)	5 (7.8)	139 (14.2)	0.19	10 (12.0)	134 (14.0)	0.74
Diabetes mellitus	203 (19.5)	14 (21.9)	189 (19.3)	0.63	22 (26.5)	181 (18.9)	0.11
Chronic liver disease	54 (5.2)	3 (4.7)	51 (5.2)	1.00	6 (7.2)	48 (5.0)	0.43
Congestive heart failure	323 (30.9)	22 (34.4)	301 (30.7)	0.58	29 (34.9)	294 (30.6)	0.46
Chronic kidney disease	94 (9.0)	5 (7.8)	89 (9.1)	1.00	5 (6.0)	89 (9.3)	0.43
Cerebrovascular disease	150 (14.3)	8 (12.5)	142 (14.5)	0.85	7 (8.4)	143 (14.9)	0.14
Malignancy	91 (8.7)	8 (12.5)	83 (8.5)	0.25	10 (12.0)	81 (8.4)	0.31
Vital signs							
Temperature, °C	37.8 (37.0–38.6)	37.1 (36.8–38.0)	38 (37.0–38.6)	0.001	38 (36.8–38.4)	38 (37.0–38.6)	0.07
Systolic blood pressure, mmHg	128 (111–148)	123 (105–143)	128(112–148)	0.21	120 (95–150)	128 (113–148)	0.032
Mean arterial pressure, mmHg	90 (78–103)	84 (76–100)	90 (78–103)	0.15	84 (69–103)	90 (78–103)	0.06
Respiratory rate, / min.	22 (20–26)	25 (20–30)	22 (19–26)	< 0.001	26 (23–30)	22 (19–25)	< 0.001
Heart rate, / min.	98 (84–111)	100 (84–115)	98 (84–111)	0.46	103 (89–124)	98 (84–110)	0.002
Mental confusion, %	134 (12.8)	24 (37.5)	110 (11.2)	< 0.001	32 (38.6)	102 (10.6)	< 0.001
Laboratory results							
Total protein, g/dl	6.6 (6.1–7.0)	6.1 (5.8–6.6)	6.6 (6.2–7.0)	< 0.001	6.1 (5.8–6.6)	6.6 (6.2–7.0)	< 0.001
Albumin, g/dl	3.2 (2.8–3.6)	2.8 (2.4–3.0)	3.3 (2.8–3.6)	< 0.001	2.9 (2.4–3.3)	3.2 (2.8–3.6)	< 0.001
AST, U/L	26 (20–39)	30 (22–44)	26 (19–39)	0.017	35 (22–58)	25 (19–37)	< 0.001
ALT, U/L	17 (12–28)	19 (11–33)	17 (12–27)	0.58	21 (14–36)	17 (12–27)	0.003
LDH, U/L	239 (195–293)	260 (211–342)	237 (195–290)	0.06	298 (240–383)	234 (193–284)	< 0.001
BUN, mg/dl	19 (14–27)	28 (20–38)	19 (14–26)	< 0.001	26 (18–41)	19 (14–25)	< 0.001
Na, mmol/L	137 (135–139)	137 (134–140)	137 (135–139)	0.90	137 (134–139)	137 (135–139)	0.35
Hgb, g/dl	12.4 (11.0–13.6)	12.2 (10.2–13.5)	12 (11.0–13.6)	0.33	13 (11.2–14.3)	12 (11.0–13.6)	0.049
WBC, × 10^9^/L	11.2 (8.1–15.2)	12.1 (8.2–15.9)	11 (8.1–15.2)	0.38	11 (7.3–15.9)	11 (8.1–15.1)	0.53
Platelets, × 10^9^/L	20.2 (15.0–26.6)	21.4 (16.4–27.6)	20 (14.9–26.5)	0.33	18 (13.3–23.7)	20 (15.3–26.9)	0.005
C-reactive protein, mg/dl	11.7 (5.5–18.8)	15.6 (11.4–24.0)	11.3 (5.2–18.3)	< 0.001	16.9 (9.3–27.9)	11.3 (5.2–17.8)	< 0.001
PCT, ng/mL	0.47 (0.14–2.22)	1.24 (0.31–6.79)	0.5 (0.13–2.11)	0.003	4.5 (0.80–17.56)	0.4 (0.13–1.84)	< 0.001
PaO_2_/FIO_2_ ratio, mmHg	265 (202–307)	178 (86–244)	267 (210–310)	< 0.001	116 (65–216)	271 (217–310)	< 0.001
PaCO_2,_ Torr	35.7 (32.0–40.2)	38.9 (31.9–52.3)	36 (32.0–40.0)	0.021	35 (30.7–47.4)	36 (32.0–40.0)	0.48
pH	7.45 (7.41–7.48)	7.4 (7.31–7.46)	7.5 (7.41–7.48)	< 0.001	7.4 (7.30–7.45)	7.5 (7.42–7.48)	< 0.001
Illness severity							
qSOFA	1 (0–1)	1 (1–2)	1 (0–1)	< 0.001	1 (1–2)	1 (0–1)	< 0.001
CURB-65	2 (1–2)	3 (2–3)	2 (1–2)	< 0.001	3 (2–4)	2 (1–2)	< 0.001
PSI points	97 (81–120)	130 (107–156)	96 (80–118)	< 0.001	128 (105–159)	96 (80–117)	< 0.001
PSI class	IV (III–IV)	IV (IV–V)	IV (III–IV)	< 0.001	IV (IV–V)	IV (III–IV)	< 0.001
SIRS	2 (1–3)	2 (1–3)	2 (1–3)	0.53	3 (2–3)	2 (1–3)	0.002
Outcomes							
Vasopressors, %	52 (5.0)	13 (20.3)	39 (4.0)	< 0.001	51 (61.4)	1 (0.1)	< 0.001
Respirator (including NPPV), %	89 (8.5)	26 (40.6)	63 (6.4)	< 0.001	69 (83.1)	20 (2.1)	< 0.001
ICU admission, %	83 (7.9)	22 (34.4)	61 (6.2)	< 0.001	–	–	
Hospital length of stay, days	11 (8–18)	11 (5–25)	11 (8–18)	0.21	21 (12–33)	11 (7–17)	< 0.001
28-day mortality, %	51 (4.9)	51 (79.7)	0 (0.0)	< 0.001	17 (20.5)	34 (3.5)	< 0.001
Hospital mortality, %	64 (6.1)	–	–		22 (26.5)	42 (4.4)	< 0.001

Data were expressed as a median (IQR) or number (%)

Abbreviations: *ALT* alanine aminotransferase, *AST* aspartate transaminase, *BUN* blood urea nitrogen, *COPD* chronic obstructive pulmonary disease, *CURB-65* confusion, urea, respiratory rate, blood pressure and age, *Hgb* hemoglobin, *ICU* intensive care unit, *IQR* interquartile range, *LDH* lactate dehydrogenase, *Na* sodium, *PSI* pneumonia severity index, *qSOFA* quick sequential (Sepsis-related) organ failure assessment, *SIRS* systemic inflammatory response syndrome, *WBC* white blood cell

The median duration of the hospital stay was 11 (8–18) days, the overall in-hospital mortality rate was 6.1%, the 28-day mortality rate was 4.9%, and the ICU admission rate was 7.9%.

### Comparison between non-survivors and survivors

The median age (80 (74–85) vs 76 (68–83) years: *p* = 0.004) was significantly higher among non-survivors than survivors. Respiratory rates (25 (20–30) / min. vs 22 (19–26) / min.: *p* < 0.001) and mental confusion (37.5 vs 11.2%: *p* < 0.001) were significantly higher among non-survivors than survivors; however, no significant differences were noted in systolic blood pressure between the two groups (*p* = 0.21).

qSOFA, CURB-65, and PSI scores were significantly higher among non-survivors than survivors (*p* < 0.001) (Additional file 1: Figure S1-a).

### Comparison between ICU and non-ICU admissions

Respiratory rates (26 (23–30) / min. vs 22 (19–25) / min.: *p* < 0.001) and mental confusion (38.6 vs 10.6%: *p* < 0.001) were significantly higher among ICU than non-ICU admissions. Systolic blood pressure was significantly lower among ICU than non-ICU admissions (120 vs 128 mmHg: *p* = 0.032).

qSOFA, CURB-65, and PSI scores were significantly higher among ICU than non-ICU admissions (*p* < 0.001) (Additional file 1: Figure S1-b).

### In-hospital mortality and ICU admission rates according to each score

Among patients with pneumonia, 13.9% (*n* = 145) were screened with sepsis based on a qSOFA score ≥ 2 points.

**Fig. 1:**
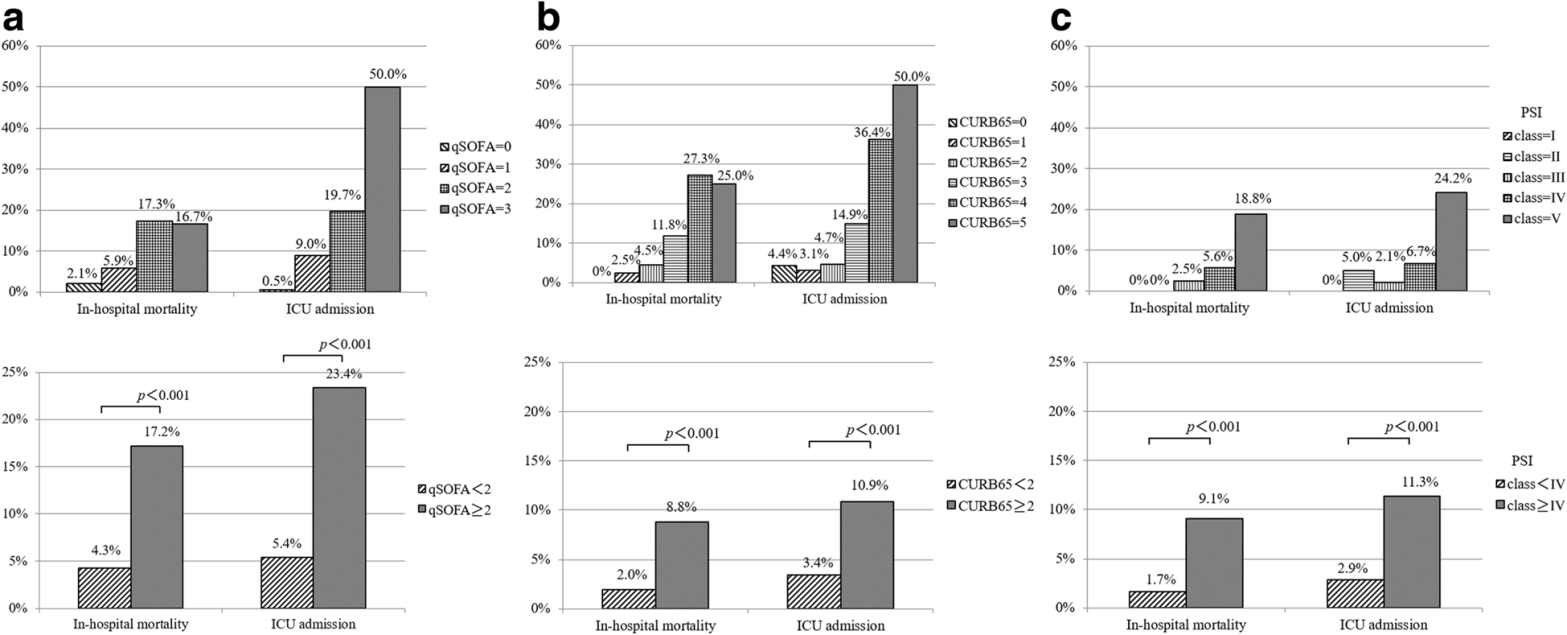
In-hospital mortality and ICU admission rates. **a** (upper) Stratified by qSOFA scores. (above) qSOFA scores less than 2 or greater than or equal to 2. **b** (upper) Stratified by CURB65 scores. (above) CURB-65 scores less than 2 or greater than or equal to 2. **c** (upper) Stratified by PSI scores. (above) PSI risk class less than IV or greater than or equal to IV. Abbreviations: *CURB-65* Confusion, Urea, Respiratory Rate, Blood Pressure and Age, *ICU* intensive care unit, *PSI* Pneumonia Severity Index, *qSOFA* quick Sequential (Sepsis-related) Organ Failure Assessment

In-hospital mortality rates were 2.1, 5.9, 17.3, and 16.7% for each of the qSOFA points. The mortality rate of a qSOFA score ≥ 2 points was significantly higher than that of a qSOFA score < 2 points (17.2 vs 4.3%, *p* < 0.001) (Fig. 1a).

ICU admission rates became higher as qSOFA scores increased. The ICU admission rate of a qSOFA score ≥ 2 points was significantly higher than that of a qSOFA score < 2 points (23.4 vs 5.4%: *p* < 0.001) (Fig. 1a).

Hospitalization was recommended for 60.8% (*n* = 635) and 59.9% (*n* = 626) of patients based on the CURB-65 score (≥ 2) and PSI class (≥ IV), respectively.

The hospital mortality and ICU admission rates of CURB-65 and PSI also became higher as qSOFA scores increased. The hospital mortality and ICU admission rates for patients with a CURB-65 score ≥ 2 points and PSI class ≥ IV were significantly higher than those with the lower cut-off values (*p* < 0.001) (Fig. 1b, c).

### Score performance

**Fig. 2:**
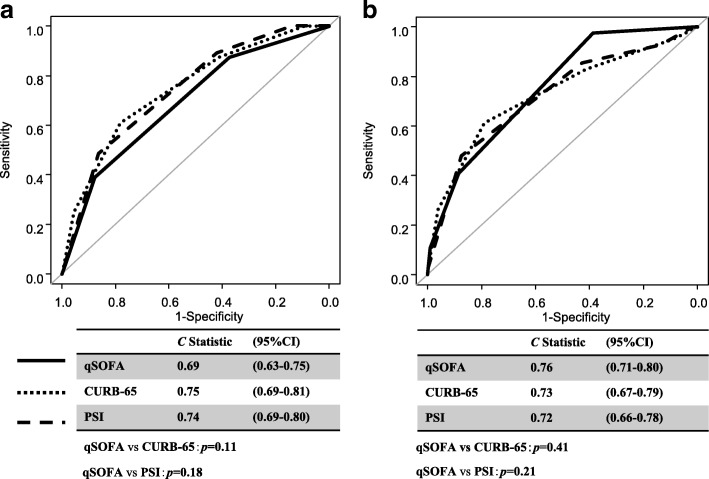
(**a**) *C* statistics for predicting hospital mortality. (**b**) *C* statistics for predicting ICU admission. Abbreviations: *CURB*-65 confusion, urea, respiratory rate, blood pressure, and age, *ICU* intensive care unit, *PSI* pneumonia severity index, *qSOFA* quick Sequential (Sepsis-related) Organ Failure Assessment

The *C* statistics of qSOFA for predicting hospital mortality was 0.69 (95% CI, 0.63–0.75), and no significant differences were observed between CURB-65 (*C* statistics, 0.75; 95% CI 0.69–0.81; *p* = 0.11) and PSI (*C* statistics, 0.74; 95% CI, 0.69–0.80; *p* = 0.18) (Fig. 2a).

The *C* statistics of qSOFA for predicting ICU admission was 0.76 (95% CI 0.71–0.80), and no significant differences were noted between CURB-65 (*C* statistics, 0.73; 95% CI 0.67–0.79; *p* = 0.41) and PSI (*C* statistics, 0.72; 95% CI 0.66–0.78; *p* = 0.21) (Fig. 2b).

### Sensitivity, specificity, and the likelihood ratio

A qSOFA score ≥ 2 points presented moderate sensitivity at 39% and high specificity at 88% for predicting hospital mortality. A CURB-65 score ≥ 2 points and PSI class ≥ IV presented high sensitivity, moderate specificity, and a high negative likelihood (Table 2).

**Table 2:** Performance of qSOFA for predicting hospital mortality

	Cut-off	Sensitivity	Specificity	PPV	NPV	OR	95%CI	
							5%	95%
qSOFA	≥ 2	39.1%	87.8%	17.2%	95.7%	4.61	2.57	8.12
CURB-65	≥ 2	87.5%	41.0%	8.8%	98.0%	4.89	2.29	12.01
PSI	≥ IV	89.1%	42.0%	9.1%	98.3%	5.88	2.64	15.46

Abbreviations: *CURB-65* Confusion, Urea, Respiratory Rate, Blood Pressure and Age; *ICU* intensive care unit; *PSI* Pneumonia Severity Index; *qSOFA* quick Sequential (Sepsis-related) Organ Failure Assessment; *PPV* positive predictive value; *NPV* negative predictive value; *OR* odds ratio

The prediction of ICU admission was similar, with a qSOFA score ≥ 2 points presenting moderate sensitivity and high specificity, and a CURB-65 score ≥ 2 points and PSI class ≥ IV presenting high sensitivity and moderate specificity (Table 3).

**Table 3:** Performance of qSOFA for predicting ICU admission

	Cut-off	Sensitivity	Specificity	PPV	NPV	OR	95%CI	
							5%	95%
qSOFA	≥ 2	41.0%	88.5%	23.4%	94.6%	5.30	3.18	8.80
CURB-65	≥ 2	83.1%	41.2%	10.9%	96.6%	3.44	1.89	6.72
PSI	≥ IV	85.5%	42.3%	11.3%	97.1%	4.33	2.29	8.90

Abbreviations: *CURB-65* Confusion, Urea, Respiratory Rate, Blood Pressure and Age; *ICU* intensive care unit; *PSI* Pneumonia Severity Index; *qSOFA* quick Sequential (Sepsis-related) Organ Failure Assessment; *PPV* positive predictive value; *NPV* negative predictive value; *OR* odds ratio

## Discussion

The present study is the first to compare the prognostic performance of qSOFA with existing pneumonia severity scores (CURB-65 and PSI) for CAP of high severity and mortality that requires hospitalization. Regarding hospitalized CAP, the prognostic performance of qSOFA for in-hospital mortality did not significantly differ from the existing pneumonia severity scores (CURB-65 and PSI). Furthermore, no significant difference was observed in the prediction performance for ICU admission. PSI is the most famous severity classification of CAP [14], and it has been clearly shown to correlate with mortality. However, this index involves 20 evaluation items and its calculation is complex; therefore, its practical use in a busy clinical setting is limited. On the other hand, CURB-65 has been described as a convenient classification with only five items (confusion, urea, respiratory rate, blood pressure, and age) with high practicality and excellent prognostic accuracy [11]. However, “Urea > 7 mmol/L” requires a blood test. qSOFA has fewer evaluation items than CURB-65 and PSI, and because it involves tests that may be conducted at the bedside, it is regarded as a simpler prognostic tool than CURB-65 and PSI.

The prognostic performance of disease-specific severity scores is excellent because the severity classification was created using a database for each disease. CURB-65 and PSI were also created from a pneumonia database [11, 14]. Therefore, it was of interest that qSOFA, which was created for general infectious diseases, did not significantly differ from pneumonia-specific severity classifications, such as CURB-65 and PSI. The use of separate severity scores for each disease is considered to be burdensome. The present results demonstrated that qSOFA, which may be used for other diseases, may be substituted for existing severity scores for CAP and may be particularly useful for non-respiratory specialists.

Three previous studies were conducted on qSOFA for CAP: 1641 patients in China (28-day mortality rate: 33%, *C* statistics for predicting 28-day mortality: 0.655), 9327 patients in Germany (30-day mortality rate: 3.0%, *C* statistics for predicting 30-day mortality: 0.70), and 6874 patients in Spain (in-hospital mortality: 6.4%, *C* statistics for predicting in-hospital mortality: 0.649) [3, 10, 12]. Although mortality rates markedly varied among these studies, the *C* statistics of qSOFA for predicting mortality ranged between 0.655 and 0.70, with no significant differences being observed between studies. While our results revealed a 28-day mortality rate of 4.9% and in-hospital mortality rate of 6.1%, the *C* statistics of 0.69 for in-hospital mortality was in accordance with previous studies [3, 10, 12]. Therefore, qSOFA may be used for CAP irrespective of mortality, regional differences, and patient background differences.

**Fig. 3:**
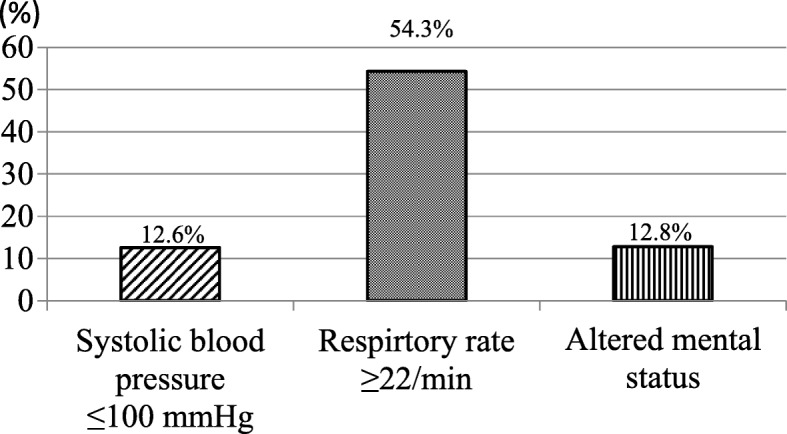
Distribution of each criterion of qSOFA. Abbreviations: *qSOFA* quick Sequential (sepsis-related) Organ Failure Assessment

The present study on patients with CAP showed that the *C* statistics of qSOFA for predicting in-hospital mortality was 0.69, which was lower than that of 0.81 in the original study [2]. Three previous studies on patients with CAP also showed *C* statistics for predicting mortality of between 0.655 and 0.70 [3, 10, 12]. The respiratory rate, which was one of the three qSOFA criteria evaluated in the present study, scored positive in at least 50% of the 1045 patients analyzed (Fig. 3). Since qSOFA only has three criteria, having a positive score for one of the criteria in more than 50% of patients may have decreased its reliability, thereby reducing the *C* statistics. The reason for the reduced *C* statistics may be the low cut-off value for the respiratory rate. Therefore, we changed the cut-off value for the respiratory rate in qSOFA to ≥ 30/min, which was the same as that in CURB-65 or PSI. However, no significant difference was observed, even though it changed from ≥ 22/min to ≥ 30/min (*C* statistics: qSOFA 0.69, qSOFA (RR ≥ 30/min) 0.71; *p* = 0.28) (Additional file 2: Figure S2).

Previous studies comparing qSOFA with CURB-65 and PSI were reviewed by the Spanish study [12] described above, in which qSOFA had a lower prognostic performance than CURB-65 and PSI. CURB-65 and PSI included age as an item of severity. The severity score in elderly patients was slightly high when measured by PSI because a patient’s age largely contributes to its scoring system. Since the median age of our study population was more than 10 years older than that of the Spanish study, patients in our study may have scored higher in PSI, thereby reducing the *C* statistics. Besides CURB-65 and PSI, many severity classifications include age as an evaluation item. Previous studies reported that it was not necessary to include age (older than 65 years) as one of the three items in qSOFA [3, 5]. In the present study, the addition of age to qSOFA did not affect the results obtained (qSOFA vs qSOFA + age ≥ 65: *p* = 0.10), although *C* statistics slightly increased to 0.71 (Additional file 3: Figure S3); therefore, the addition of age was unnecessary based on convenience.

In the present study, 13.9% of pneumonia cases were diagnosed with sepsis with a qSOFA score ≥2 points, and the mortality rate of a qSOFA score ≥ 2 points was 17.2%. This severity was close to “life-threatening organ dysfunction caused by a dysregulated host response to infection, in-hospital mortality rate 10%” defined as sepsis-3. As the score of qSOFA became higher, in-hospital mortality and ICU admission rates gradually increased, the in-hospital mortality rate for patients with a qSOFA score ≥ 2 points was fourfold higher than those with lower cut-off values, and the ICU admission rate was fivefold higher. Previous studies that investigated qSOFA in CAP showed high specificity, but moderate sensitivity, similar to the present study. On the other hand, CURB-65 and PSI showed high sensitivity and moderate specificity. *C* statistics were no significantly different between these predictive models; qSOFA might be used to screen severe pneumonia, because of its high specificity.

### Limitations

The limitations of the present study were as follows. First, it was a single-center secondary analysis of data from a prospective observational study. The prospective observational study of qSOFA was only one study targeting all infectious diseases in the emergency department [4], and there has not yet been a prospective observational study on qSOFA for pneumonia. Therefore, multicenter prospective studies are needed in order to validate our results. Furthermore, although the *C* statistics of qSOFA for predicting mortality was similar to the three previous studies [3, 10, 12], there are still only four studies on pneumonia, including the present study; therefore, the evaluation of qSOFA for pneumonia is currently insufficient. In addition, it is unclear whether qSOFA may be used reliably for patients in different regions or with varying backgrounds. Second, it was that the present study only considered hospitalized CAP. Pneumonia in patients with a poor performance status and hospital-acquired pneumonia also need to be considered. Third, the present study have the potential for the information bias given our retrospective study design. We attempted to reduce the information bias by using a prospectively collected data. Forth, the decision to enter the ICU is decided based on preset ICU admission criteria (such as, respiratory failure requiring mechanical ventilation, circulatory failure requiring vasopressor, confusion, etc.). However, potential selection bias existed, e.g., the decision of the physician might be biased by the score. Finally, if the period from emergency department visit to ICU admission was long, the meaning of the score at entry could be diminished. But in our study, the length from emergency department visit to ICU admission was all within 7 days except for two cases.

## Conclusion

Regarding hospitalized CAP, the prognostic performance of qSOFA for in-hospital mortality and ICU admission was not significantly different to those of CURB-65 and PSI. Since qSOFA only requires a few items and vital signs, it may be particularly useful for emergency department or non-respiratory specialists.

## Additional files

Additional file 1: **Figure S1.** Distribution of illness severity. **a** Survivors and non-survivors. **b** Non-ICU admission and ICU admission. (PPTX 111 kb)

Additional file 2: **Figure S2.** *C* statistics for predicting hospital mortality (qSOFA [RR ≥ 30]). (PPTX 77 kb)

Additional file 3: **Figure S3.** *C* statistics for predicting hospital mortality (qSOFA + age ≥ 65). (PPTX 76 kb)

## Abbreviations

CURB-65: Confusion, urea, respiratory rate, blood pressure, and age
ICU: Intensive care unit
PSI: Pneumonia Severity Index
qSOFA: Quick Sequential (sepsis-related) Organ Failure Assessment
