# Supplementary material for: The prognostic performance of qSOFA for community-acquired pneumonia
Source: J Intensive Care. 2018 Aug 8;6:46. doi: 10.1186/s40560-018-0307-7 (PMC6083584; doi:10.1186/s40560-018-0307-7)
Supplement: Supplementary file 1 — Figure S1. Distribution of illness severity. a Survivors and non-survivors. b Non-ICU admission and ICU admission. (PPTX 111 kb) [file 40560_2018_307_MOESM1_ESM.pptx]

## Slide 1
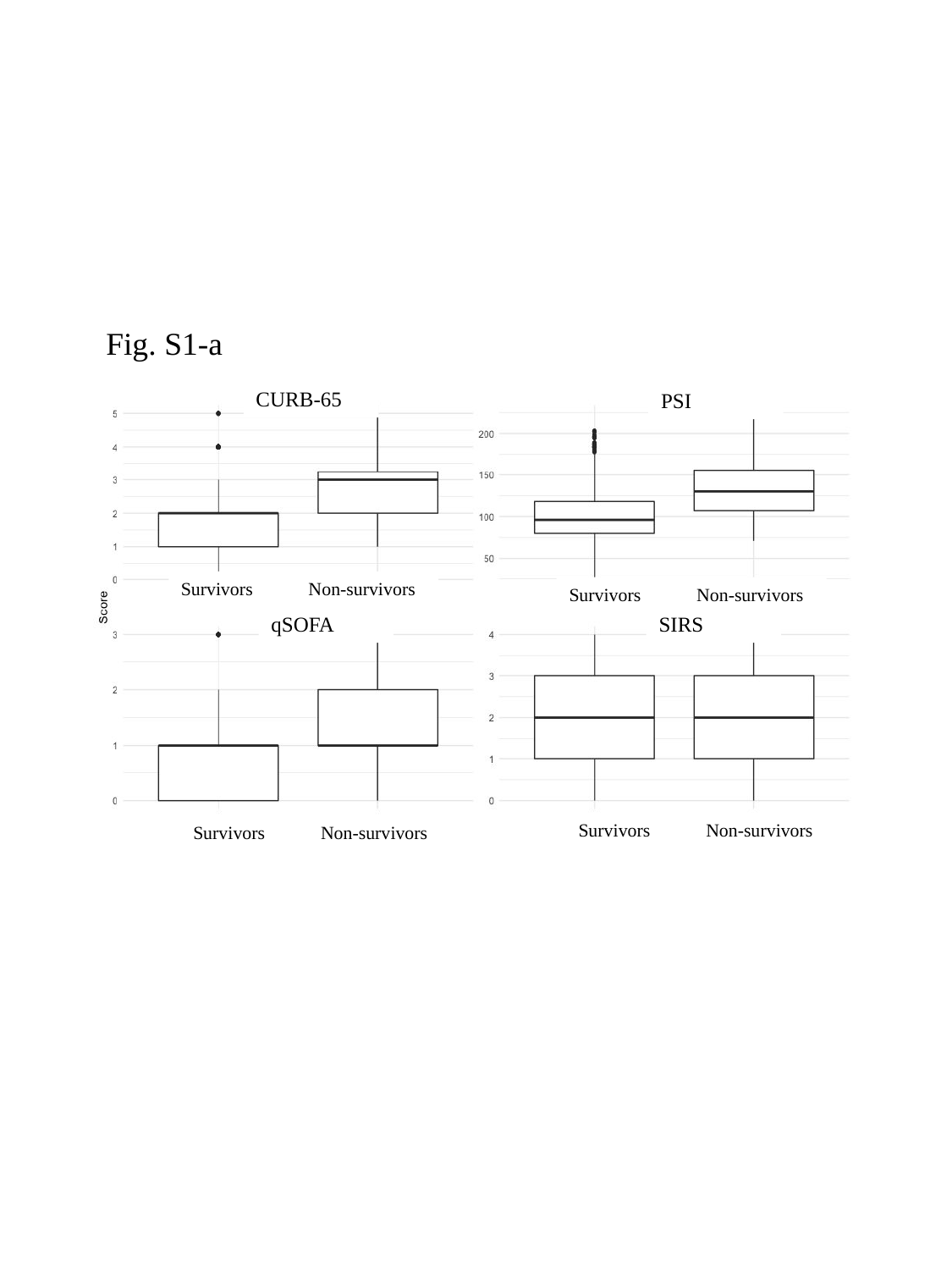

Fig. S1-a
CURB-65
PSI
Survivors Non-survivors
Survivors Non-survivors
qSOFA
SIRS
Survivors Non-survivors
 Survivors Non-survivors

## Slide 2
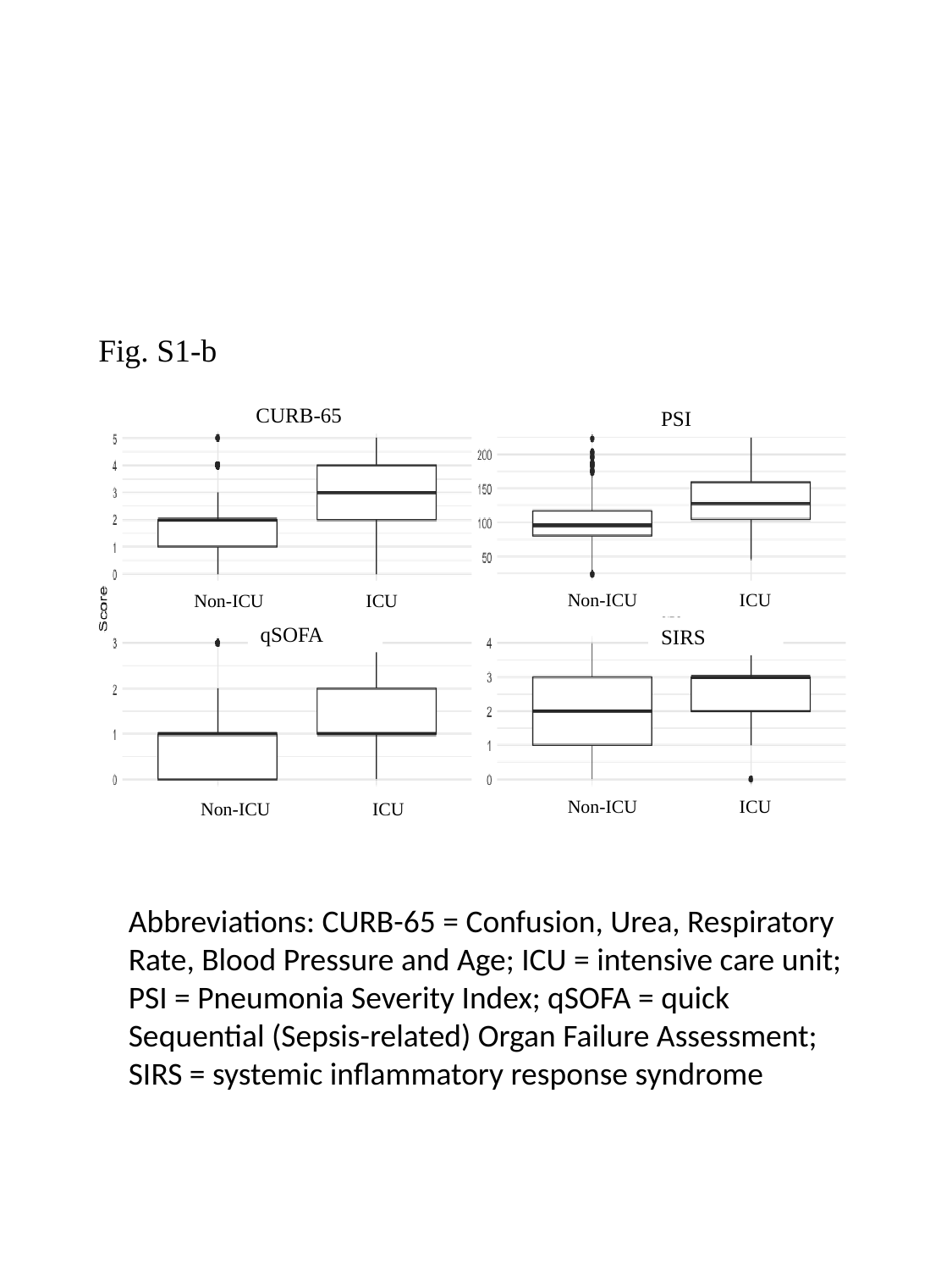

Fig. S1-b
CURB-65
PSI
Non-ICU ICU
Non-ICU ICU
qSOFA
SIRS
Non-ICU ICU
Non-ICU ICU
Abbreviations: CURB-65 = Confusion, Urea, Respiratory Rate, Blood Pressure and Age; ICU = intensive care unit; PSI = Pneumonia Severity Index; qSOFA = quick Sequential (Sepsis-related) Organ Failure Assessment; SIRS = systemic inflammatory response syndrome
