# Supplementary material for: The prognostic performance of qSOFA for community-acquired pneumonia
Source: J Intensive Care. 2018 Aug 8;6:46. doi: 10.1186/s40560-018-0307-7 (PMC6083584; doi:10.1186/s40560-018-0307-7)
Supplement: Supplementary file 3 — Figure S3. C statistics for predicting hospital mortality (qSOFA + age ≥ 65). (PPTX 76 kb) [file 40560_2018_307_MOESM3_ESM.pptx]

## Slide 1
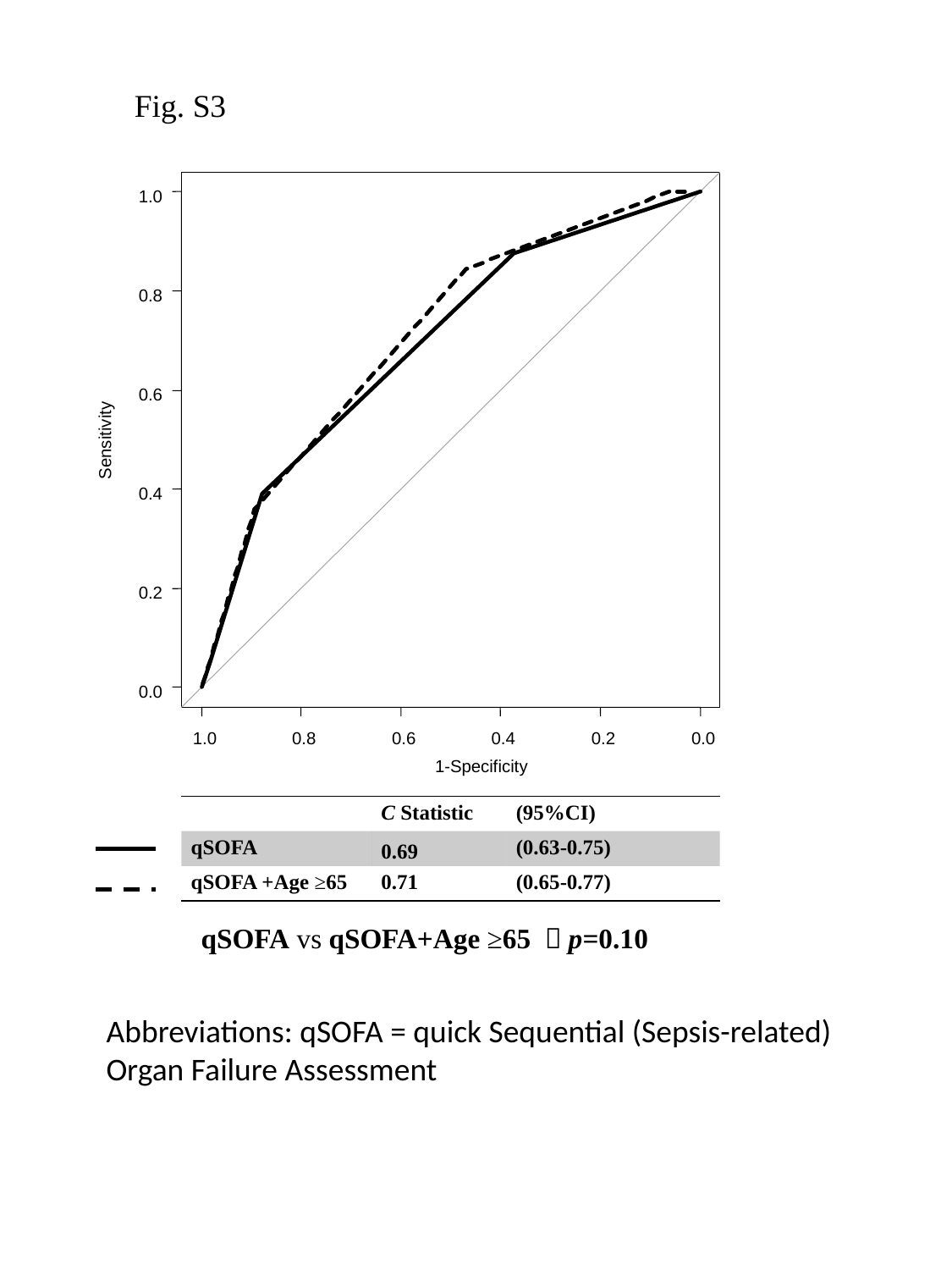

Fig. S3
1.0
0.8
0.6
Sensitivity
0.4
0.2
0.0
1.0
0.8
0.6
0.4
0.2
0.0
1-Specificity
| | C Statistic | (95%CI) |
| --- | --- | --- |
| qSOFA | 0.69 | (0.63-0.75) |
| qSOFA +Age ≥65 | 0.71 | (0.65-0.77) |
qSOFA vs qSOFA+Age ≥65 ：p=0.10
Abbreviations: qSOFA = quick Sequential (Sepsis-related) Organ Failure Assessment
